# Nematode infection in liver of the fish *Gymnotus inaequilabiatus* (Gymnotiformes: Gymnotidae) from the Pantanal Region in Brazil: pathobiology and inflammatory response

**DOI:** 10.1186/s13071-016-1772-2

**Published:** 2016-08-30

**Authors:** Bahram Sayyaf Dezfuli, Carlos E. Fernandes, Gizela M. Galindo, Giuseppe Castaldelli, Maurizio Manera, Joseph A. DePasquale, Massimo Lorenzoni, Sara Bertin, Luisa Giari

**Affiliations:** 1 Department of Life Sciences and Biotechnology, University of Ferrara, Ferrara, Italy; 2 Laboratory of Pathology, CCBS, Federal University of Mato Grosso do Sul, Campo Grande, Brazil; 3 Department of Food Science, University of Teramo, St. Crispi 212, 64100 Teramo, Italy; 4 Morphogenyx Inc, PO Box 717, 11731 East Northport, NY USA; 5 Department of Cellular and Environmental Biology, University of Perugia, St. Elce di Sotto 5, 06123 Perugia, Italy

**Keywords:** Fish immune response, Nematode larvae, Hepatic granuloma, Histopathology

## Abstract

**Background:**

A survey on endoparasitic helminths from freshwater fishes in the Pantanal Region (Mato Grosso do Sul, Brazil) revealed the occurrence of third-larval stage of the nematode *Brevimulticaecum* sp. (Heterocheilidae) in most organs of *Gymnotus inaequilabiatus* (Gymnotidae) also known by the local name tuvira. The aim of the present study was to examine *Brevimulticaecum* sp.-infected tuvira liver at the ultrastructural level and clarify the nature of granulomas and the cellular elements involved in the immune response to nematode larvae.

**Methods:**

Thirty-eight adult specimens of tuvira from Porto Morrinho, were acquired in January and March 2016. Infected and uninfected liver tissues were fixed and prepared for histological and ultrastructure investigations.

**Results:**

The prevalence of infection of tuvira liver by the nematode larvae was 95 %, with an intensity of infection ranging from 4 to 343 larvae (mean ± SD: 55.31 ± 73.94 larvae per liver). In livers with high numbers of nematode larvae, almost entire hepatic tissue was occupied by the parasites. Hepatocytes showed slight to mild degenerative changes and accumulation of pigments. Parasite larvae were surrounded by round to oval granulomas, the result of focal host tissue response to the infection. Each granuloma was typically formed by three concentric layers: an outer layer of fibrous connective tissue with thin elongated fibroblasts; a middle layer of mast cells entrapped in a thin fibroblast-connective mesh; and an inner layer of densely packed epithelioid cells, displaying numerous desmosomes between each other. Numerous macrophage aggregates occurred in the granulomas and in the parenchyma.

**Conclusions:**

Our results in tuvira showed that the larvae were efficiently sequestered within the granulomas, most of the inflammatory components were confined within the thickness of the granuloma, and the parenchyma was relatively free of immune cells and without fibrosis. Presumably this focal encapsulation of the parasites permits uninfected portions of liver to maintain its functions and allows the survival of the host.

## Background

Ten species of the genus *Brevimulticaecum* (Nematoda: Heterocheilidae) have been reported from different parts of the world, of which six species are exclusively distributed in the Americas [1]. Species of this genus have an indirect and complex life-cycle, which involves intermediate, paratenic and definitive hosts. Some macroinvertebrates (e.g. aquatic insects such as members of Odonata and Ephemeroptera) are intermediate hosts of *Brevimulticaecum* sp. [2] and amphibians, snakes and freshwater fish have a role as paratenic hosts while crocodilians are definitive hosts [3–7].

Encapsulated third-stage larvae of several species of nematodes are commonly found in the organs of marine [8–12] and freshwater fish [13–16]. A survey on endoparasitic helminths from freshwater fishes in the Pantanal Region (Mato Grosso do Sul, Brazil) revealed the occurrence of third-stage larvae of *Brevimulticaecum* sp. in most organs of *Gymnotus inaequilabiatus* [1, 2, 17]. *Gymnotus inaequalabiatus*, also known by the local name tuvira, is of great importance in southern Brazil as it is used for live-bait to collect other fish species of commercial value. Tuvira is a preferred prey for the Pantanal caiman, *Caiman yacare* [18] and adult specimens of *Brevimulticaecum* sp. were recorded from the intestine of this reptile [3].

In intestine and visceral organs of most fish species, larvae of helminths often become encapsulated by a host tissue response [13, 19, 20], with the walls of the capsule consisting of thick connective tissue and epithelioid cells [21, 22]. The basic immune response mechanisms in vertebrates against helminths are poorly understood [23]. In response to infection, a variety of cells become activated and cooperate in an effort to control and eliminate the invading pathogens [24]. In fish, the innate defences responding to helminth infection commonly involve mast cells (MCs) [20, 25–27], macrophage aggregates (MAs) or melano-macrophage centres [22, 27, 28], neutrophils [29, 30] and rodlet cells (RCs) [27, 31].

In a recent study on the histopathology of tuvira liver infected with *Brevimulticaecum* sp. Ventura et al. [17] stated that “The lack of acute inflammatory process indicates that the parasite is not causing to the host a reaction with inflammatory cells …”. However their study was based on light microscopy which is not sufficient for an accurate determination of liver infection by *Brevimulticaecum* sp. While histopathology can be used to assess the health impact of parasitism [32], it is well established that ultrastructural observation analysis of the liver is a superior tool for determining health status [33]. The aim of the present study was to examine *Brevimulticaecum* sp.-infected tuvira liver at the ultrastructural level and clarify the nature of granulomas as well as the cellular elements involved in the immune response to nematode larvae.

## Methods

Adult specimens (*n* = 38) of *Gymnotus inaequilabiatus*, from Porto Morrinho (21°41′56″S, 57°52′57″W), municipality of Corumbá, Brazil, were collected in January and March 2016. The specimens were transported in oxygenated polyethylene bags to the Laboratory of Pathology, Federal University of Mato Grosso do Sul, Brazil, where fishes were stocked for two hours in aquariua supplied with artificial aeration at constant temperature (25 °C) until euthanasia (2-Fenoxiethanol, 2 ml/l). Euthanized fish were then weighed (total weight in grams) and measured (total length in centimeters). After dissection stomach, intestine, liver, heart, gonads, spleen, and kidney were screened microscopically for endoparasites. The liver was removed from the coelomic cavity and although cysts were visible with a naked eye, a more accurate assessment was made by examining the organ surface under a stereomicroscope. From each infected liver, several 10 × 10 mm pieces were excised and fixed in 10 % neutral buffered formalin for 24 h, then processed routinely for paraffin embedding, sectioned (5 μm thick) and then stained with either Hematoxylin and Eosin, alcian blue 8 GX pH 2.5 and periodic acid Schiff’s (AB/PAS), Perls’ Prussian Blue stain for ferric iron, Giemsa and/or Masson’s Trichrome. Multiple histological sections were taken from each tissue block, examined and photographed using an optical microscope (Nikon Eclipse 80i; Nikon, Tokyo, Japan).

For transmission electron microscopy (TEM), 7 × 7 mm pieces of infected liver tissues were fixed in chilled 2.5 % glutaraldehyde in 0.1 M sodium cacodylate buffer for 3 h. The fixed tissues were then post-fixed in 1 % osmium tetroxide for 2 h and then rinsed and stored in 0.1 M sodium cacodylate buffer containing 6 % sucrose for 12 h. Thereafter, the tissue pieces were dehydrated through a graded acetone series and embedded in epoxy resin (Durcupan ACM, Fluka, Buchs, Switzerland). Semi-thin sections (1.5 μm) were cut on a Reichert Om U 2 ultramicrotome and stained with toluidine blue. Ultra-thin sections (90 nm) were stained with 4 % uranyl acetate solution in 50 % ethanol and Reynold’s lead citrate and then examined using an Hitachi H-800 transmission electron microscope.

For both light and transmission electron microscopy corresponding pieces of uninfected liver were also processed so that a direct comparison with the infected material could be made. From ten livers, over 40 encysted larvae were isolated and fixed in acetic acid and stored in ethanol 70 % and then cleared in lactophenol for species identification.

## Results

**Fig. 1:**
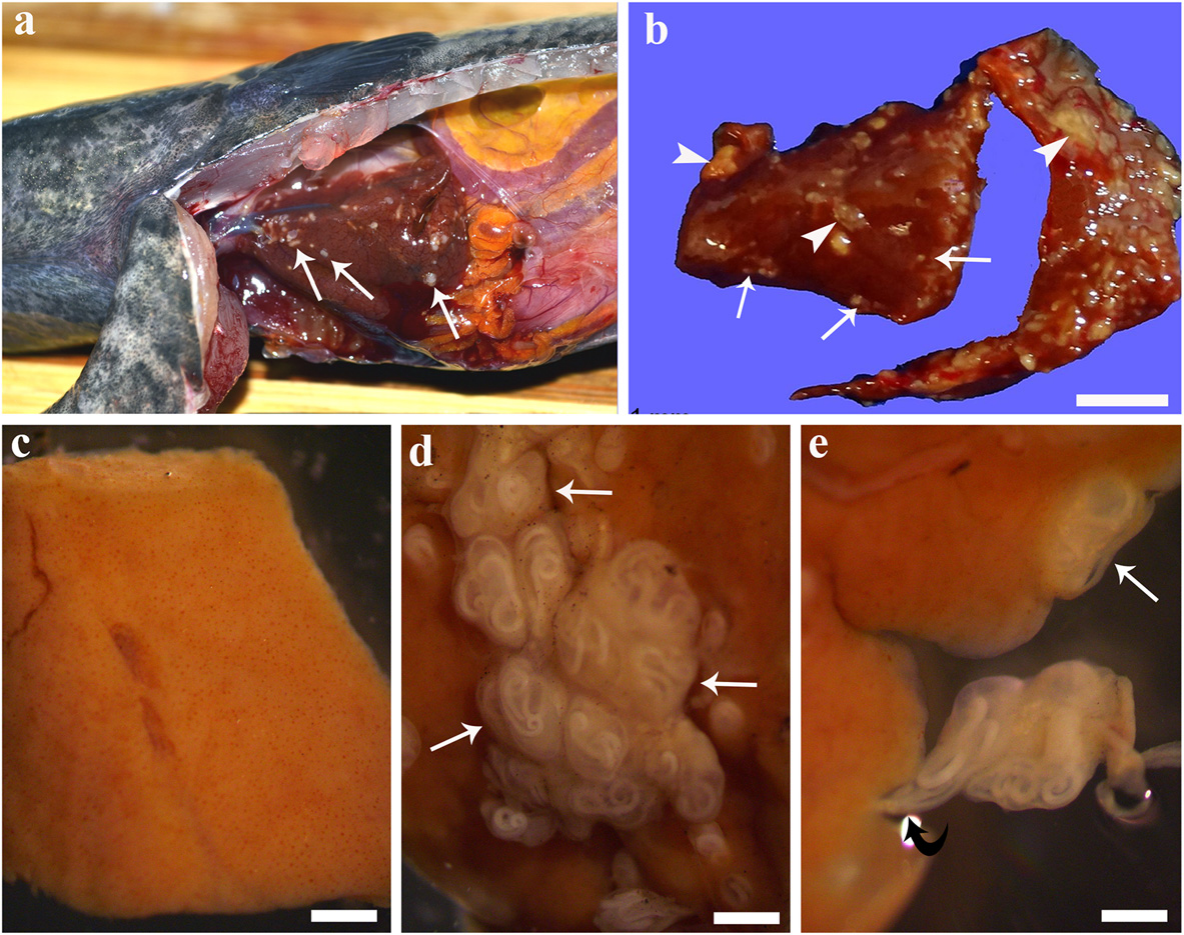
**a** Photograph during necropsy; several encysted larvae (*arrows*) of the nematode *Brevimulticaecum* sp. are visible on liver of *Gymnotus inaequilabiatus.* **b** Very heavily infected liver shown during necropsy examination; the larva appeared as single (*arrows*) or in clusters (*arrowheads*) beneath the serosa. **c** Small piece of fixed liver free of larvae; smooth surface of organ is appreciable. **d** Cluster of *Brevimulticaecum* sp. third-stage larva in cysts (*arrows*) covered by serosa are evident. Note the difference between surfaces of liver in (**c** and **d**). **e** Encysted larva on surface of liver (arrow), and numerous larvae inside the cyst attached to the liver by a peduncle (*curved arrow*). *Scale-bars*: **b**, 1 mm; **c**, 2 mm; **d**, 2 mm; **e**, 1.5 mm

Thirty-eight specimens of *G. inaequilabiatus* (mean total length ± standard deviation, SD: 32.36 ± 2.89 cm) were sampled and examined. Nematode larvae were encountered in almost all of the visceral organs of *G. inaequilabiatus*, but liver appeared to be the most infected organ (Fig. 1a, b). Ninety-five per cent of livers (36 of 38) were infected with nematode larvae (Fig. 1a, b). Based on morphological examinations of a sub-population of larvae isolated/removed from the liver, all specimens were identified as third-stage larvae of *Brevimulticaecum* sp. based on key features provided in Moravec & Kaiser [4] and Moravec et al. [34]. The range in intensity of infection was 4–343 larvae per fish (mean ± SD: 55.31 ± 73.94 larvae per liver). Figure 1c shows a small piece of uninfected liver. In infected liver, the vast majority of the larvae was located either singly or as an aggregation of white cysts beneath the serosa of the liver (Fig. 1b, d, e) and within the hepatic parenchyma. Large cysts containing white *Brevimulticaecum* sp. larvae were noticeable by naked eye during necropsy examination (Fig. 1a, b). In many instances, single larvae or clusters of larvae were observed to be attached to the liver surface by a kind of peduncle of host peritoneal origin (Fig. 1e).

**Fig. 2:**
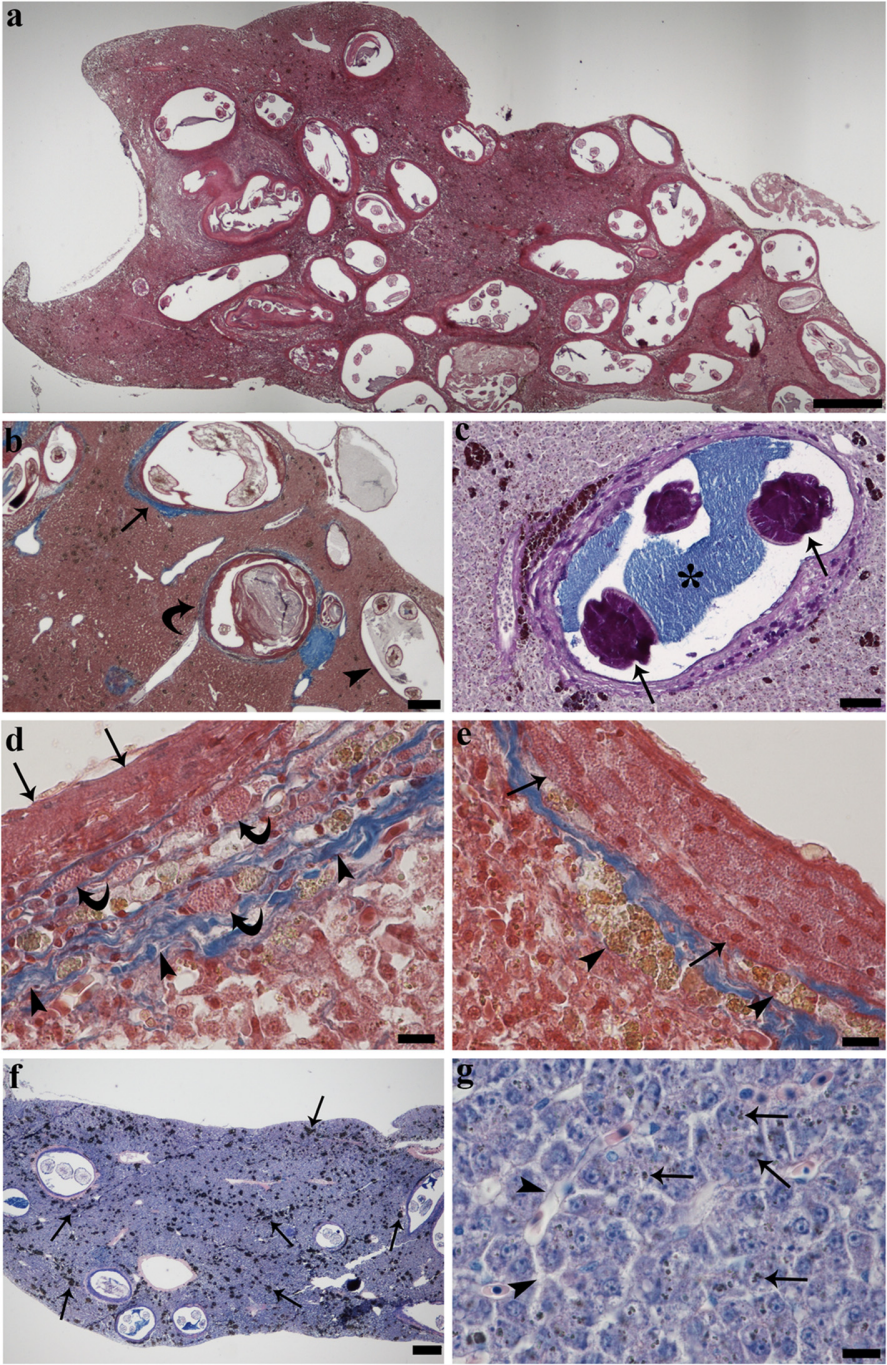
**a** Sagittal section of heavily infected liver; the cysts are round to oval in shape and likely occupy the whole hepatic surface (hematoxylin & eosin). **b** Three different granulomatous reactions are apparent. The granuloma on the right (*arrowhead*) lacks an evident collagenous encapsulation, which is present in the other two granulomas (*arrow* and *curved arrow*). The presence of necrotic material exfoliating form the inner layer is clearly appreciable in the granuloma in the middle of the figure (*curved arrow*) (Masson’s Trichrome). **c** Inside the cyst the nematode (*arrows*) is immersed in an Alcian Blue positive fluid (*asterisk*) (Alcian Blue-PAS). **d** Stratification of granuloma is visible, inner layer formed by epithelioid cells (*arrows*), middle layer is constituted by mast cells (*curved arrows*), outer layer with connective fibres (*arrowheads*) and elongated fibroblasts (Masson’s Trichrome). **e** Infected liver; intense degranulation of the mast cells (*arrows*) and macrophage aggregates (*arrowheads*) are evident (Masson’s Trichrome). **f** Sagittal section of the infected liver; numerous macrophage aggregates are evident (*arrows*) (Giemsa). **g** Sinusoids dilation (*arrowheads*) and liver hydropic degeneration are appreciable, brownish pigments are visible inside the liver cells (*arrows*) (Giemsa). *Scale-bars*: **a**, 500 μm; **b**, f, 200 μm; **c**, 50 μm; **d**, **e**, 10 μm; **g**, 10 μm

In cases of heavy infection, the vast majority of liver surface was occupied by larvae (Fig. 2a). *Brevimulticaecum* sp. larvae were encircled by focal host tissue reaction, forming round-oval shaped granulomas (Fig. 2a-c). In most granulomas, especially in those with larger larvae, exfoliation of the inner layer of epithelioid cells resulted in accumulation of amorphous necrotic tissue (Fig. 2b). This necrotic accumulation was slightly to strongly basophilic and PAS positive. Where this exfoliation and related necrosis were absent or moderately present, the cysts were enlarged by means of a faintly eosinophilic, Alcian Blue positive fluid (Fig. 2c). Granulomas were formed by three concentric layers: an outer layer of fibrous connective tissue and thin elongated fibroblasts (Fig. 2d); a middle layer of MCs (Fig. 2d) entrapped in a thin fibroblastic-connective mesh, usually spongiotic in appearance; and an inner layer of densely-packed epithelioid cells (Fig. 2d). The thickness of each layer varied considerably among granulomas (Fig. 2b). MCs showed intense PAS and slightly Alcian Blue pH 2.5 positive granules. Degranulation of MCs was frequent (Fig. 2e).

A net separation was clearly visible between the fibrous connective layer and the remnant tissue; moreover, fibrous connective branches within liver septa were seen in larger granulomas. Nevertheless, there was no evidence of true liver fibrosis. Infected liver had numerous MAs (Fig. 2f). MAs occurred as focal and large accumulation of macrophages (see further) laden with brownish pigments (melanin, chromolipoids, hemosiderin) within the thickness of the granulomas as well as scattered in the parenchyma (Fig. 2e, f). Furthermore, the presence of amorphous proteinaceous amphoterous faintly PAS-positive material, was detected among the connective fibres. Sinusoids dilation, liver hydropic degeneration, and occurrence of brownish pigments (mainly hemosiderin and, to a lesser extent, lipofuscin) were visible inside hepatocytes in parasitized liver (Fig. 2g).

**Fig. 3:**
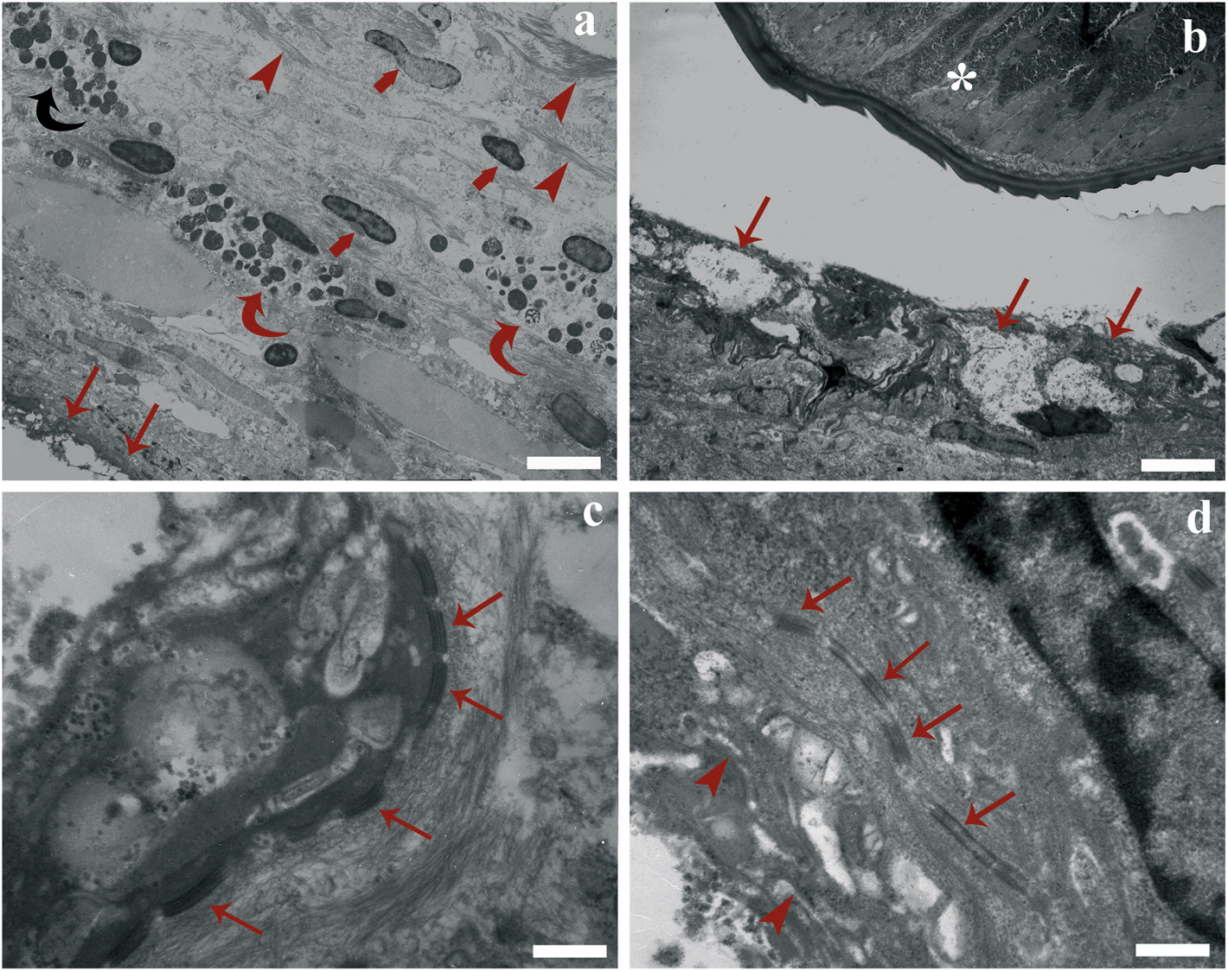
**a** Transmission electron microscope micrograph showing three layers which formed the granuloma; inner layer formed by epithelioid cells (*arrows*), mast cells (*curved arrows*) of the middle layer, fibroblasts (*thick arrows*) and connective fibres (*arrowheads*) of the outer layers. **b** The epithelioid cells (*arrows*) surrounding the nematode larva (*asterisk*); note the foamy aspect of the epithelioid cells. **c** Numerous desmosomes (*arrows*) between epithelioid cells are visible. **d** Interdigitation (*curved arrows*) and desmosomes (*arrows*) between the epithelioid cells. *Scale-bars*: **a**, 3.64 μm; **b**, 3.57 μm; **c**, 0.26 μm; **d**, 0.25 μm

Ultrastructural observations revealed the features of the cell types in each layer of the granuloma (Fig. 3a). The inner layer appeared darker (Fig. 3b, c) than the middle and outer layer of granulomas. The innermost layer of cells surrounding the nematode larvae was composed of elongated transformed macrophages, namely epithelioid cells (Fig. 3a-d). Their nuclei were rich in euchromatin and their cytoplasm contained many filaments, free ribosomes and swollen mitochondria. Numerous desmosomes between epithelioid cells (Fig. 3c, d) and some interdigitations between these cells were noted (Fig. 3d). In some instances, the epithelioid cells displayed a foamy appearance (Fig. 3b).

**Fig. 4:**
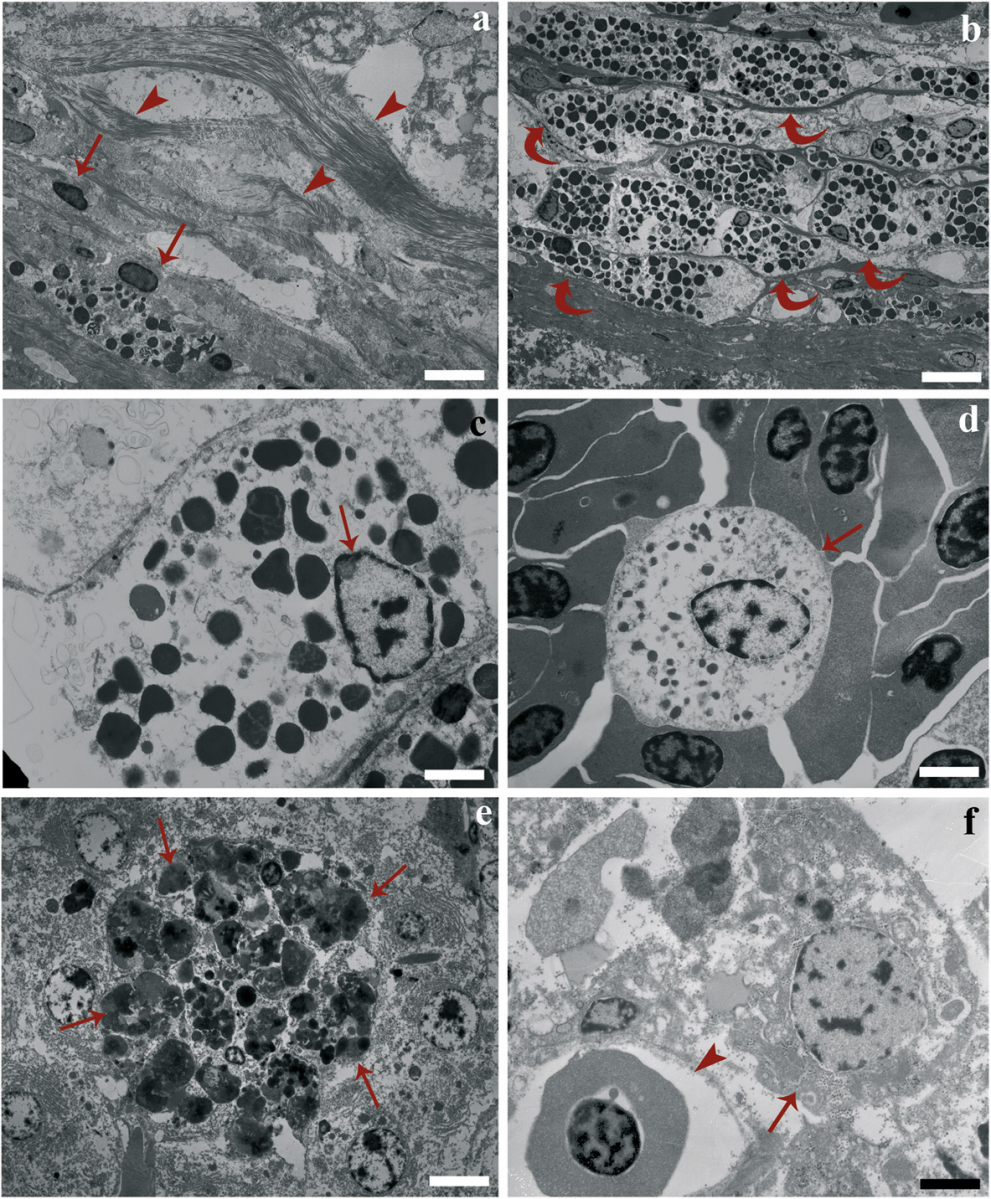
**a** Micrograph showing the outer layer of the granuloma, abundant collagenous fibres (*arrowheads*) and fibroblasts (*arrows*). **b** High magnification of the middle layer; note many mast cells (*curved arrows*) and electron-dense granules in their cytoplasm. **c** Eccentric nucleus (*arrow*) and several electron-dense granules of a mast cells. **d** A neutrophil (*arrow*) surrounded with blood cells within the lumen of a hepatic sinusoid. **e** Macrophage aggregates (MAs) formed by groups of large oval to round cells (*arrows*) inside liver parenchyma. **f** A hepatocyte (*arrow*) appeared as large polyhedral cell in close proximity to a small vessel (*arrowhead*). *Scale-bars*: **a**, 3.33 μm; **b**, 5.00 μm; **c**, 1.30 μm; **d**, 1.75 μm; **e**, 4.36 μm; **f**, 1.55 μm

Figure 4a shows the outer layer of the granuloma with numerous collagenous fibres and some fibroblasts. In the middle layer, numerous granular cells were noticed (Fig. 4b) encircling the epithelioid corona. They contained numerous polymorphic dense granules that displaced the nucleus to the periphery (Fig. 4c) and were identified as MCs. The cytoplasm typically contained two to three mitochondria as well as several electron-dense granules (Fig. 4b, c). Frequently high numbers of MCs increased the thickness of the middle layer (Fig. 4b). Degranulation of the MCs was observed in many granulomas near the nematode larva. Consistent with this degranulation, the matrix of the granules had an extensively reticulated aspect (not shown). Another type of a granular cell, the neutrophil, was found scattered among the blood cells in sinusoid lumen (Fig. 4d), as well as in the parenchymal interstitium. The neutrophils contained smaller granules than those of MCs. These granules were rod-shaped and possessed an elongated, electron dense, lamellar core (not shown).

With reference to the rodlet cells (RCs), their number was low and they were occasionally observed in the epithelium of the biliary ducts and rarely associated with sinusoids. In a few infected livers RCs also occurred in the parenchymal interstitium, near the encysted larva. MAs associated with the nematode larvae consisted of groups of large oval to round cells (Fig. 4e) with eccentric polar nuclei with marked peripheral accumulations of chromatin. The cytoplasm of these cells at low magnification appeared partially vacuolated and foamy, and contained inclusions of differing electron-densities (not shown).

In infected livers, hepatocytes appeared as large polyhedral cells (Fig. 4f), with large and pale euchromatic nuclei, prominent nucleoli, partial enlargement of the rough endoplasmic reticulum cisternae, mitochondria dilation and focal vacuolisation (not shown). Focal cytoplasm rarefaction was also appreciable. The histological and ultrastructural liver pattern was suggestive of slight to mild hepatocyte hydropic degeneration.

## Discussion

The nematode *Brevimulticaecum* sp. has been reported in several freshwater fish species (e.g. *G. inaequalabiatus*) in the Pantanal wetland in Barzil [1]. All internal organs of tuvira were infected with third larval stage of *Brevimulticaecum* sp. However the very high intensity of nematode infection in liver induced us to focus on this organ in order to accurately assess the damage to hepatic tissue.

Nematode parasites harm their host in different ways, such as causing mechanical injury [35, 36], atrophy of tissues, impairment of host fecundity [37], and pathogenecity of liver [9, 13, 17]. Parasitic helminths in natural habitats can drastically reduce their host fitness, and accordingly the hosts have evolved powerful counter-measures to control infection [38]. It is reasonable to concur with the statement of Secombes & Chappell [39] and Franke et al. [40] that the successful infection of helminths largely depends on their capacity to evade and/or manipulate the generally efficient immune system of hosts. In recent years there has been a renaissance in the study of fish immune responses [27, 41–43], which significantly expanded our knowledge of the evolution and diversification of the vertebrate immune system [44, 45]. Innate immunity in teleosts relies on a range of cell types [46]. Below we will examine in turn each immune cell type involved in the response of tuvira liver to *Brevimulticaecum* sp. larvae.

MCs exist in all classes of vertebrates, sharing both a similar morphology and, most likely, function [47]. MCs are motile [25, 48] and are often strategically positioned at perivascular sites to regulate inflammation, to encounter invading organisms and to orchestrate a response [27, 42, 48–51]. MCs degranulate in response to a variety of pathogens [50, 52] and known degranulating agents [42, 53]. Degranulation of fish MCs close to the tegument of helminths in intestine and other organs was reported in Dezfuli et al. [50, 54]. In the present study degranulation of MCs in close proximity to the cuticle of *Brevimulticaecum* sp. was observed in numerous granulomas. MCs play an important role in responding to inflammation [41] and their numbers increase as a consequence of helminth infection in fish intestine [20, 27, 55] as well as in parasitized liver [13, 22, 56]. Numerous MCs were noticed to be in close contact with capillaries and the outer layer of the endothelia as well as within the lumen of the blood vessels in an infected organ [27, 57, 58]. Similar findings were found in liver and pancreas of *Phoxinus phoxinus* infected with larvae of the nematode *Raphidascaris acus* [13], consistent with *G. inaequilabiatus* liver harbouring *Brevimulticaecum* sp. larvae observed here. MCs are involved in the fibrotic process and in tissue remodelling [22, 55, 59]. The results of the current study suggest that *Brevimulticaecum* sp. larva in the tuvira liver preferentially induces the recruitment of MCs, fibroblasts and other immune cells (e.g. macrophages and epithelioid cells) to sites of infection. Moreover, we believe that MCs association with fibroblasts and macrophages may mediate liver remodelling/repair after extensive tissue injury caused by the nematode larva.

Phagocytosis is a well-conserved innate defense mechanism with phagocytes contributing to both pro-inflammatory and anti-inflammatory (resolution) responses at infectious foci [52, 60, 61]. Two major professional phagocyte populations have been described in fish: granulocytes (particularly neutrophils) and mononuclear phagocytes (circulating monocytes and tissue macrophages) [46, 62]. Neutrophils have an important role in the inflammatory process, especially during the period of initial pathogen challenge, migrating from the blood to accumulate at the site of injury or parasitic infection [20, 29, 30, 46, 54]. As mentioned in the previous section, neutrophils were observed in the parenchyma, hepatic sinusoid lumen, and/or interstitium around the capillary in infected tuvira liver. Surprisingly, no neutrophils were observed within the thickness of the granuloma nor in close proximity to the *Brevimulticaecum* sp. cuticle. In contrast, neutrophils were observed within the capsule surrounding the larva of *R. acus* that had infected *P. phoxinus* liver [13].

Macrophages have emerged as an essential cell type in all vertebrates, endowed with a panoply of capacities [63, 64]. Within an inflammatory site, macrophages are exposed to both pro-inflammatory stimuli and dying cells [60]. Fish macrophages contain different types of pigments including melanin, chromolipoids and hemosiderin [46, 65], consequently, these groups of cells are named macrophage aggregates (MAs) or melano-macrophage centres [28, 66]. MAs may be found within tissue encapsulating many foreign bodies and parasites [20, 66, 67]. MAs functions have been reported to include the focal destruction, detoxification and recycling of endogenous and exogenous materials [68, 69]. Accordingly, MAs are involved against helminth infection [20, 22, 28, 65]. In infected liver of tuvira where numerous MAs were observed there was no recognizable distribution pattern, and in several cases MAs were found within the thickness of the granuloma wall and occasionally within the inner layer and very close to the larval body. Despite the occurrence of numerous neutrophils and MAs in parasitized liver we do not have direct evidence that these phagocytes killed the larva.

The inner layer of the capsule which surrounds *Brevimulticaecum* sp. larva was formed by epithelioid cells. These cells are so-named because of their morphological similarity to epithelial cells, and indeed are considered differentiated, transformed macrophages that form with persistent inflammatory stimulation [70, 71]. The formation of epithelioid cells in fish is known to occur from a variety of insults and their origin from macrophages was observed in vitro [72]. The transition from metabolically active cells (i.e. macrophages and, to a lesser extent, epithelioid cells) appears to be very interesting aspect of liver response to form a structural sequestering element.

Only occasional RCs were present within the epithelium of the bile ducts of tuvira liver infected with nematode larvae. Data on numerous fish-helminth systems suggest that RCs represent an inflammatory cell type closely linked to other piscine immune cells (e.g. MCs and epithelioid cells) [13, 20, 25, 27, 31].

Some studies on cod liver from the Baltic Sea revealed a remarkable increase of up to 100 % in the prevalence of infection with nematodes [11, 73]. It was suggested that high infection of cod livers by parasites is associated with the successful re-introduction of grey seals (definitive hosts) in the Baltic Sea [74]. The present study also noted a high prevalence of infection (95 %) with *Brevimulticaecum* sp. larva in the liver of tuvira. This fish is one of the most preferred preys for *Caiman yacare* in the study area. This crocodilian acts as a definitive host for several helminth species (e.g. *Brevimulticaecum* sp.) [3] and in the past was extensively harvested for its skin, illegally through direct hunting, and legally through ranching and collecting wild eggs [75], but in the last decade is considered a protected species [18]. Similarly to the grey seals of the Baltic Sea, it is reasonable to presume that high numbers of *C. yacare* in the Pantanal Region ensure to keep very high the prevalence of *Brevimulticaecum* sp. in the liver of fish paratenic host (e.g. tuvira).

## Conclusions

The present study utilized histopathological and ultrastructural assays to provide evidence that pathobiology relies entirely on a focal encapsulating reaction. In heavily infected livers most of the organ is occupied by the encysted parasites and associated enveloping granulomas. However the less extensive regions of uninfected parenchyma were relatively free of immune cells, without fibrosis and showed only slight to mild structural damage to hepatocytes. In other host-nematode systems, the encircling reaction closely resembled that described above for tuvira, although the liver status may differ particularly close to the inflammatory foci (see [13, 76]). Despite the high prevalence and intensity of infection of *Brevimulticaecum* sp. in tuvira there appears to be little risk of decline of the fish population, presumably thanks to the balance between parasite damage, host defence response and liver compensatory properties.

## Abbreviations

AB/PAS: Alcian blue/periodic acid Schiff
MAs: Macrophage aggregates
MCs: Mast cells
PAS: Periodic acid Schiff
RCs: Rodlet cells
SD: Standard deviation
TEM: Transmission electron microscopy
